# 17β-Hy­droxy-17α-(hy­droxy­meth­yl)estr-4-en-3-one

**DOI:** 10.1107/S160053681004033X

**Published:** 2010-10-23

**Authors:** Sammer Yousuf, Salman Zafar, Muhammed Iqbal Choudhary, Seik Weng Ng

**Affiliations:** a H.E.J. Research Institute of Chemistry, International Center for Chemical and Biological Sciences, University of Karachi, Karachi 75270, Pakistan; b Department of Chemistry, University of Malaya, 50603 Kuala Lumpur, Malaysia

## Abstract

The title compound, C_19_H_28_O_3_, the fungal-transformed metabolite of the steroid methyl­oestrenol contains four fused rings *A*, *B*, *C* and *D*. Ring *A* adopts a half-chair and the *trans*-fused rings *B* and *C* adopt chair confirmations; the five-membered *D* ring is folded like an envelope. In the crystal, adjacent mol­ecules are linked by O—H⋯O_carbon­yl_ and O—H⋯O_hy­droxy_ hydrogen bonds into a layer structure.

## Related literature

For the synthesis, see: Hübner & Ponsold (1983 ▶); Ponsold *et al.* (1978*a* ▶,*b* ▶); Szilagyi *et al.* (1984 ▶). For the crystal structures of three modified17*b*-hy­droxy-3-oxo-17*a*-(halogen/pseudo­halogenometh­yl)-estra-4-ene progestagens, see: Beck *et al.* (1986*a* ▶,*b* ▶,*c* ▶).
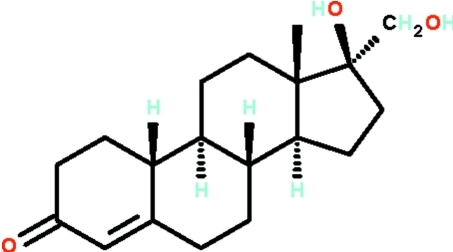


## Experimental

### 

#### Crystal data

C_19_H_28_O_3_
*M* *_r_* = 304.41
Orthorhombic, 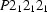

*a* = 9.9696 (6) Å
*b* = 12.5858 (8) Å
*c* = 13.3968 (8) Å
*V* = 1680.97 (18) Å^3^
*Z* = 4
Mo *K*α radiation
μ = 0.08 mm^−1^
*T* = 295 K
0.35 × 0.20 × 0.10 mm

#### Data collection

Bruker SMART APEX CCD diffractometer
11685 measured reflections
2203 independent reflections
1799 reflections with *I* > 2σ(*I*)
*R*_int_ = 0.047

#### Refinement

*R*[*F* ^2^ > 2σ(*F* ^2^)] = 0.047
*wR*(*F* ^2^) = 0.119
*S* = 1.11
2203 reflections
207 parameters
2 restraints
H atoms treated by a mixture of independent and constrained refinement
Δρ_max_ = 0.19 e Å^−3^
Δρ_min_ = −0.28 e Å^−3^

### 

Data collection: *SMART* (Bruker, 2003 ▶); cell refinement: *SAINT* (Bruker, 2003 ▶); data reduction: *SAINT*; program(s) used to solve structure: *SHELXS97* (Sheldrick, 2008 ▶); program(s) used to refine structure: *SHELXL97* (Sheldrick, 2008 ▶); molecular graphics: *X-SEED* (Barbour, 2001 ▶); software used to prepare material for publication: *publCIF* (Westrip, 2010 ▶).

## Supplementary Material

Crystal structure: contains datablocks global, I. DOI: 10.1107/S160053681004033X/hb5675sup1.cif

Structure factors: contains datablocks I. DOI: 10.1107/S160053681004033X/hb5675Isup2.hkl

Additional supplementary materials:  crystallographic information; 3D view; checkCIF report

## Figures and Tables

**Table 1:** Hydrogen-bond geometry (Å, °)

*D*—H⋯*A*	*D*—H	H⋯*A*	*D*⋯*A*	*D*—H⋯*A*
O2—H2⋯O3^i^	0.84 (3)	1.95 (3)	2.779 (3)	171 (3)
O3—H3⋯O1^ii^	0.84 (3)	2.18 (3)	2.904 (3)	144 (4)

Symmetry codes: (i) 
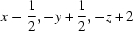
; (ii) 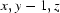.

**Fig. 1.**
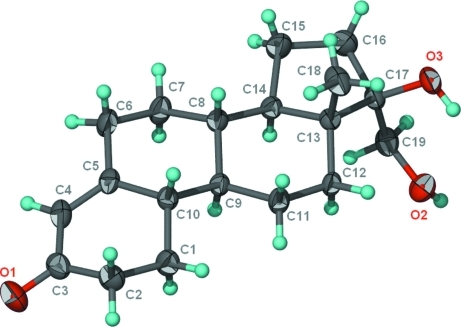
The molecuylar structure of (I) at the 50% probability level; hydrogen atoms are drawn as spheres of arbitrary radius.

**Fig. 2.**
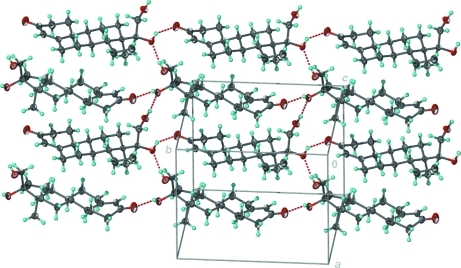
Layer structure.

## supplementary crystallographic information

### Comment

Structure modification by using microbes, cells and fungi, *i.e.*, biotransfromation, is an excellent method for understanding the structure-activity relationship of bioactive compounds and drugs. In this study, a new compound was isolated when the estrogen drug methyloestrenolone was incubated with *Aspergillus niger*. This study represents the first report of the biotransformation of methyloestrenolone; the compound is 17α-(hydroxymethyl)-estr-4-en-17β-ol-3-one (Scheme I, Fig. 1). Adjacent molecules are linked by *O*–*H*···O_carbonyl_ and *O*–*H*···O_hydroxy_ hydrogen bonds into a layer structure (Fig. 2). Bond dimensions are similar to those found in three other 17β-hydroxy-3-oxo-17α-(halogen/pseudohalogenomethyl)-estra-4-ene progestagens (Beck *et al.*, 1986*a*,*b*,*c*).

The title compound has been obtained by conventional chemical synthesis (Hübner & Ponsold, 1983; Ponsold *et al.*, 1978*a*, 1978*b*; Szilagyi *et al.*, 1984).

### Experimental

*Culture preparation*

In 4 *L* water were dissolved glucose (40 g), peptone (20 g), yeast extract (12 g), potassium dihydrogen phosphate (20 g), sodium chloride (20 g) and glycerol (40 ml). The solution was distributed among 40 conical flasks (100 ml each); the mouths of the flasks were covered with cotton wool. The flasks were then heated at 374 K for 15 minutes. The spores of *Aspergillus nige*r were transferred from slants grown on saboraud dextrose agar. The flasks were left on rotary shaker until there was sufficient growth of the spores. Methyloestrenolone (1 g) was distributed equally among the flasks in the form of its solution in acetone (20 ml, 0.5 ml per flask).

*Fermentation of methyloestrenolone*

Methyloestrenolone was also incubated with a liquid phase culture of *Aspergillus niger* (4 *L*) for 14 days. The biomass was separated by filtration and the filtrate extracted with dichloromethane The extract was dried with sodium sulfate; the solvent was evaporated to leave about 3 g of a brown gummy material. This was subjected to fractionation on a silica gel column with petroleum ether–ethyl acetate gradient solvent system. The fractions were subjected to size exclusion HPLC (GS-320, methanol, 35 minute retention time). Evaporation of the solvent gave the title compound as colorless prisms of (I).

### Refinement

Carbon-bound H-atoms were placed in calculated positions (C—H 0.93 to 0.98 Å) and were included in the refinement in the riding model approximation, with *U*(H) set to 1.2 to 1.5*U*(C).

The hydroxy H-atoms were located in a difference Fourier map, and were refined with a distance restraint of O–H 0.84±0.01 Å; their temperature factors were freely refined.

The absolute configuration was assumed to be that of methyloestrenolone itself; 1656 Friedel pairs were merged.

### Crystal data

**Table:**

C_19_H_28_O_3_	*F*(000) = 664
*M**_r_* = 304.41	*D*_x_ = 1.203 Mg m^−^^3^
Orthorhombic, *P*2_1_2_1_2_1_	Mo *K*α radiation, λ = 0.71073 Å
Hall symbol: P 2ac 2ab	Cell parameters from 2043 reflections
*a* = 9.9696 (6) Å	θ = 2.5–21.3°
*b* = 12.5858 (8) Å	µ = 0.08 mm^−^^1^
*c* = 13.3968 (8) Å	*T* = 295 K
*V* = 1680.97 (18) Å^3^	Prism, colorless
*Z* = 4	0.35 × 0.20 × 0.10 mm

### Data collection

**Table:**

Bruker SMART APEX CCD diffractometer	1799 reflections with *I* > 2σ(*I*)
Radiation source: fine-focus sealed tube	*R*_int_ = 0.047
graphite	θ_max_ = 27.5°, θ_min_ = 2.2°
ω scans	*h* = −12→12
11685 measured reflections	*k* = −15→16
2203 independent reflections	*l* = −17→13

### Refinement

**Table:**

Refinement on *F*^2^	Primary atom site location: structure-invariant direct methods
Least-squares matrix: full	Secondary atom site location: difference Fourier map
*R*[*F*^2^ > 2σ(*F*^2^)] = 0.047	Hydrogen site location: inferred from neighbouring sites
*wR*(*F*^2^) = 0.119	H atoms treated by a mixture of independent and constrained refinement
*S* = 1.11	*w* = 1/[σ^2^(*F*_o_^2^) + (0.0591*P*)^2^ + 0.0486*P*] where *P* = (*F*_o_^2^ + 2*F*_c_^2^)/3
2203 reflections	(Δ/σ)_max_ = 0.001
207 parameters	Δρ_max_ = 0.19 e Å^−^^3^
2 restraints	Δρ_min_ = −0.28 e Å^−^^3^

### Fractional atomic coordinates and isotropic or equivalent isotropic displacement parameters (Å^2^)

**Table:**

	*x*	*y*	*z*	*U*_iso_*/*U*_eq_	
O1	0.5961 (3)	1.11961 (17)	1.13914 (18)	0.0682 (7)	
O2	0.39264 (19)	0.32100 (16)	1.04677 (15)	0.0492 (5)	
O3	0.6378 (2)	0.26561 (14)	0.97416 (16)	0.0462 (5)	
C1	0.6147 (3)	0.8388 (2)	1.17666 (19)	0.0464 (7)	
H1A	0.5176	0.8385	1.1722	0.056*	
H1B	0.6408	0.7906	1.2297	0.056*	
C2	0.6631 (3)	0.9507 (2)	1.2017 (2)	0.0506 (7)	
H2A	0.6235	0.9734	1.2643	0.061*	
H2B	0.7598	0.9503	1.2097	0.061*	
C3	0.6256 (3)	1.0271 (2)	1.1211 (2)	0.0451 (6)	
C4	0.6340 (3)	0.9862 (2)	1.0191 (2)	0.0420 (6)	
H4	0.6185	1.0328	0.9666	0.050*	
C5	0.6628 (2)	0.88515 (19)	0.99789 (19)	0.0356 (6)	
C6	0.6928 (3)	0.8510 (2)	0.89256 (19)	0.0438 (7)	
H6A	0.7893	0.8480	0.8839	0.053*	
H6B	0.6584	0.9044	0.8471	0.053*	
C7	0.6336 (3)	0.74360 (19)	0.86388 (19)	0.0445 (7)	
H7A	0.6689	0.7220	0.7995	0.053*	
H7B	0.5370	0.7499	0.8579	0.053*	
C8	0.6673 (3)	0.65901 (18)	0.94171 (18)	0.0333 (5)	
H8	0.7651	0.6532	0.9468	0.040*	
C9	0.6117 (3)	0.69403 (18)	1.04371 (16)	0.0310 (5)	
H9	0.5153	0.7064	1.0350	0.037*	
C10	0.6743 (3)	0.80063 (18)	1.07782 (17)	0.0329 (5)	
H10	0.7701	0.7879	1.0890	0.040*	
C11	0.6269 (3)	0.60786 (18)	1.12419 (19)	0.0393 (6)	
H11A	0.7209	0.6015	1.1418	0.047*	
H11B	0.5785	0.6298	1.1835	0.047*	
C12	0.5748 (3)	0.49914 (18)	1.09091 (16)	0.0342 (6)	
H12A	0.5940	0.4470	1.1423	0.041*	
H12B	0.4782	0.5026	1.0826	0.041*	
C13	0.6389 (2)	0.46399 (18)	0.99316 (17)	0.0306 (5)	
C14	0.6106 (3)	0.55026 (19)	0.91475 (17)	0.0336 (5)	
H14	0.5129	0.5584	0.9116	0.040*	
C15	0.6536 (4)	0.4988 (2)	0.8161 (2)	0.0542 (8)	
H15A	0.7488	0.5087	0.8044	0.065*	
H15B	0.6040	0.5285	0.7604	0.065*	
C16	0.6199 (3)	0.3807 (2)	0.8303 (2)	0.0525 (7)	
H16A	0.6973	0.3373	0.8140	0.063*	
H16B	0.5466	0.3605	0.7867	0.063*	
C17	0.5796 (2)	0.36350 (19)	0.94063 (18)	0.0343 (5)	
C18	0.7903 (3)	0.4452 (2)	1.0088 (2)	0.0494 (7)	
H18B	0.8291	0.5060	1.0412	0.074*	
H18C	0.8329	0.4345	0.9454	0.074*	
H18D	0.8033	0.3835	1.0498	0.074*	
C19	0.4282 (3)	0.3522 (2)	0.9490 (2)	0.0419 (6)	
H19A	0.3857	0.4195	0.9331	0.050*	
H19B	0.3968	0.2996	0.9016	0.050*	
H2	0.3200 (19)	0.288 (2)	1.041 (2)	0.065 (10)*	
H3	0.595 (4)	0.242 (3)	1.023 (2)	0.098 (16)*	

### Atomic displacement parameters (Å^2^)

**Table:**

	*U*^11^	*U*^22^	*U*^33^	*U*^12^	*U*^13^	*U*^23^
O1	0.0825 (17)	0.0374 (11)	0.0847 (16)	0.0077 (11)	0.0139 (13)	−0.0098 (11)
O2	0.0399 (11)	0.0470 (11)	0.0606 (13)	−0.0140 (9)	0.0059 (9)	0.0023 (9)
O3	0.0407 (11)	0.0276 (9)	0.0702 (15)	0.0056 (8)	0.0070 (10)	0.0029 (9)
C1	0.0666 (19)	0.0341 (14)	0.0384 (14)	−0.0083 (13)	0.0058 (13)	−0.0056 (11)
C2	0.064 (2)	0.0374 (14)	0.0500 (17)	−0.0081 (14)	0.0078 (15)	−0.0113 (12)
C3	0.0394 (14)	0.0333 (14)	0.0627 (17)	−0.0052 (12)	0.0067 (13)	−0.0055 (12)
C4	0.0438 (15)	0.0303 (13)	0.0520 (15)	−0.0035 (11)	−0.0025 (13)	0.0058 (11)
C5	0.0342 (13)	0.0307 (12)	0.0418 (14)	−0.0074 (10)	0.0000 (11)	0.0038 (10)
C6	0.0580 (17)	0.0341 (14)	0.0395 (14)	−0.0086 (13)	0.0047 (12)	0.0086 (11)
C7	0.0672 (19)	0.0362 (14)	0.0300 (13)	−0.0089 (14)	−0.0019 (13)	0.0038 (10)
C8	0.0377 (12)	0.0302 (12)	0.0319 (12)	−0.0034 (10)	0.0021 (10)	0.0003 (9)
C9	0.0335 (12)	0.0270 (11)	0.0326 (12)	−0.0035 (10)	0.0008 (10)	0.0013 (9)
C10	0.0375 (13)	0.0287 (12)	0.0325 (13)	−0.0033 (10)	−0.0022 (10)	−0.0002 (9)
C11	0.0582 (17)	0.0310 (12)	0.0288 (12)	−0.0027 (12)	−0.0022 (12)	0.0035 (9)
C12	0.0461 (15)	0.0276 (12)	0.0291 (12)	−0.0033 (11)	0.0013 (10)	0.0052 (9)
C13	0.0276 (12)	0.0288 (11)	0.0354 (13)	−0.0004 (10)	0.0002 (10)	−0.0006 (9)
C14	0.0392 (13)	0.0312 (12)	0.0305 (12)	−0.0036 (11)	0.0015 (10)	−0.0007 (9)
C15	0.085 (2)	0.0407 (15)	0.0373 (15)	−0.0107 (16)	0.0157 (15)	−0.0060 (12)
C16	0.071 (2)	0.0397 (15)	0.0470 (16)	−0.0072 (15)	0.0116 (15)	−0.0112 (13)
C17	0.0348 (12)	0.0263 (12)	0.0417 (13)	0.0010 (10)	0.0006 (11)	−0.0003 (10)
C18	0.0350 (15)	0.0390 (15)	0.074 (2)	0.0006 (12)	−0.0044 (14)	−0.0035 (14)
C19	0.0380 (13)	0.0345 (14)	0.0532 (15)	−0.0042 (11)	−0.0071 (12)	0.0012 (12)

### Geometric parameters (Å, °)

**Table:**

O1—C3	1.225 (3)	C9—C10	1.549 (3)
O2—C19	1.413 (3)	C9—H9	0.9800
O2—H2	0.84 (3)	C10—H10	0.9800
O3—C17	1.434 (3)	C11—C12	1.530 (3)
O3—H3	0.84 (3)	C11—H11A	0.9700
C1—C2	1.526 (4)	C11—H11B	0.9700
C1—C10	1.529 (3)	C12—C13	1.523 (3)
C1—H1A	0.9700	C12—H12A	0.9700
C1—H1B	0.9700	C12—H12B	0.9700
C2—C3	1.493 (4)	C13—C14	1.537 (3)
C2—H2A	0.9700	C13—C18	1.543 (3)
C2—H2B	0.9700	C13—C17	1.563 (3)
C3—C4	1.463 (4)	C14—C15	1.533 (3)
C4—C5	1.334 (4)	C14—H14	0.9800
C4—H4	0.9300	C15—C16	1.536 (4)
C5—C6	1.505 (4)	C15—H15A	0.9700
C5—C10	1.514 (3)	C15—H15B	0.9700
C6—C7	1.524 (3)	C16—C17	1.546 (4)
C6—H6A	0.9700	C16—H16A	0.9700
C6—H6B	0.9700	C16—H16B	0.9700
C7—C8	1.527 (3)	C17—C19	1.520 (3)
C7—H7A	0.9700	C18—H18B	0.9600
C7—H7B	0.9700	C18—H18C	0.9600
C8—C14	1.524 (3)	C18—H18D	0.9600
C8—C9	1.539 (3)	C19—H19A	0.9700
C8—H8	0.9800	C19—H19B	0.9700
C9—C11	1.537 (3)		
			
C19—O2—H2	106 (2)	C12—C11—H11A	108.9
C17—O3—H3	110 (3)	C9—C11—H11A	108.9
C2—C1—C10	111.0 (2)	C12—C11—H11B	108.9
C2—C1—H1A	109.4	C9—C11—H11B	108.9
C10—C1—H1A	109.4	H11A—C11—H11B	107.8
C2—C1—H1B	109.4	C13—C12—C11	111.6 (2)
C10—C1—H1B	109.4	C13—C12—H12A	109.3
H1A—C1—H1B	108.0	C11—C12—H12A	109.3
C3—C2—C1	110.9 (2)	C13—C12—H12B	109.3
C3—C2—H2A	109.5	C11—C12—H12B	109.3
C1—C2—H2A	109.5	H12A—C12—H12B	108.0
C3—C2—H2B	109.5	C12—C13—C14	107.80 (19)
C1—C2—H2B	109.5	C12—C13—C18	109.8 (2)
H2A—C2—H2B	108.1	C14—C13—C18	112.4 (2)
O1—C3—C4	122.2 (3)	C12—C13—C17	117.6 (2)
O1—C3—C2	122.0 (3)	C14—C13—C17	101.23 (18)
C4—C3—C2	115.7 (2)	C18—C13—C17	107.9 (2)
C5—C4—C3	123.2 (3)	C8—C14—C15	118.7 (2)
C5—C4—H4	118.4	C8—C14—C13	113.9 (2)
C3—C4—H4	118.4	C15—C14—C13	103.9 (2)
C4—C5—C6	121.0 (2)	C8—C14—H14	106.5
C4—C5—C10	122.3 (2)	C15—C14—H14	106.5
C6—C5—C10	116.6 (2)	C13—C14—H14	106.5
C5—C6—C7	114.4 (2)	C14—C15—C16	103.9 (2)
C5—C6—H6A	108.7	C14—C15—H15A	111.0
C7—C6—H6A	108.7	C16—C15—H15A	111.0
C5—C6—H6B	108.7	C14—C15—H15B	111.0
C7—C6—H6B	108.7	C16—C15—H15B	111.0
H6A—C6—H6B	107.6	H15A—C15—H15B	109.0
C6—C7—C8	111.2 (2)	C15—C16—C17	108.1 (2)
C6—C7—H7A	109.4	C15—C16—H16A	110.1
C8—C7—H7A	109.4	C17—C16—H16A	110.1
C6—C7—H7B	109.4	C15—C16—H16B	110.1
C8—C7—H7B	109.4	C17—C16—H16B	110.1
H7A—C7—H7B	108.0	H16A—C16—H16B	108.4
C14—C8—C7	112.5 (2)	O3—C17—C19	107.4 (2)
C14—C8—C9	109.51 (19)	O3—C17—C16	108.3 (2)
C7—C8—C9	109.1 (2)	C19—C17—C16	110.0 (2)
C14—C8—H8	108.5	O3—C17—C13	113.66 (19)
C7—C8—H8	108.5	C19—C17—C13	114.7 (2)
C9—C8—H8	108.5	C16—C17—C13	102.65 (19)
C11—C9—C8	112.66 (19)	C13—C18—H18B	109.5
C11—C9—C10	111.39 (19)	C13—C18—H18C	109.5
C8—C9—C10	111.39 (19)	H18B—C18—H18C	109.5
C11—C9—H9	107.0	C13—C18—H18D	109.5
C8—C9—H9	107.0	H18B—C18—H18D	109.5
C10—C9—H9	107.0	H18C—C18—H18D	109.5
C5—C10—C1	111.2 (2)	O2—C19—C17	110.1 (2)
C5—C10—C9	111.69 (19)	O2—C19—H19A	109.6
C1—C10—C9	111.8 (2)	C17—C19—H19A	109.6
C5—C10—H10	107.3	O2—C19—H19B	109.6
C1—C10—H10	107.3	C17—C19—H19B	109.6
C9—C10—H10	107.3	H19A—C19—H19B	108.2
C12—C11—C9	113.1 (2)		
			
C10—C1—C2—C3	58.7 (3)	C11—C12—C13—C18	65.7 (3)
C1—C2—C3—O1	145.9 (3)	C11—C12—C13—C17	−170.4 (2)
C1—C2—C3—C4	−37.6 (4)	C7—C8—C14—C15	59.1 (3)
O1—C3—C4—C5	−178.4 (3)	C9—C8—C14—C15	−179.4 (2)
C2—C3—C4—C5	5.1 (4)	C7—C8—C14—C13	−178.2 (2)
C3—C4—C5—C6	−169.7 (3)	C9—C8—C14—C13	−56.7 (3)
C3—C4—C5—C10	7.0 (4)	C12—C13—C14—C8	60.2 (3)
C4—C5—C6—C7	−140.6 (3)	C18—C13—C14—C8	−60.9 (3)
C10—C5—C6—C7	42.5 (3)	C17—C13—C14—C8	−175.7 (2)
C5—C6—C7—C8	−50.0 (3)	C12—C13—C14—C15	−169.2 (2)
C6—C7—C8—C14	−179.5 (2)	C18—C13—C14—C15	69.7 (3)
C6—C7—C8—C9	58.8 (3)	C17—C13—C14—C15	−45.2 (2)
C14—C8—C9—C11	50.0 (3)	C8—C14—C15—C16	161.0 (2)
C7—C8—C9—C11	173.5 (2)	C13—C14—C15—C16	33.4 (3)
C14—C8—C9—C10	175.97 (19)	C14—C15—C16—C17	−8.6 (3)
C7—C8—C9—C10	−60.5 (3)	C15—C16—C17—O3	−139.3 (3)
C4—C5—C10—C1	14.6 (4)	C15—C16—C17—C19	103.6 (3)
C6—C5—C10—C1	−168.6 (2)	C15—C16—C17—C13	−18.8 (3)
C4—C5—C10—C9	140.3 (2)	C12—C13—C17—O3	−87.4 (3)
C6—C5—C10—C9	−42.9 (3)	C14—C13—C17—O3	155.4 (2)
C2—C1—C10—C5	−46.5 (3)	C18—C13—C17—O3	37.3 (3)
C2—C1—C10—C9	−172.2 (2)	C12—C13—C17—C19	36.6 (3)
C11—C9—C10—C5	178.6 (2)	C14—C13—C17—C19	−80.5 (2)
C8—C9—C10—C5	51.9 (3)	C18—C13—C17—C19	161.3 (2)
C11—C9—C10—C1	−56.0 (3)	C12—C13—C17—C16	155.8 (2)
C8—C9—C10—C1	177.2 (2)	C14—C13—C17—C16	38.7 (2)
C8—C9—C11—C12	−50.1 (3)	C18—C13—C17—C16	−79.5 (3)
C10—C9—C11—C12	−176.1 (2)	O3—C17—C19—O2	53.7 (3)
C9—C11—C12—C13	54.1 (3)	C16—C17—C19—O2	171.4 (2)
C11—C12—C13—C14	−56.9 (3)	C13—C17—C19—O2	−73.6 (3)

### Hydrogen-bond geometry (Å, °)

**Table:**

*D*—H···*A*	*D*—H	H···*A*	*D*···*A*	*D*—H···*A*
O2—H2···O3^i^	0.84 (3)	1.95 (3)	2.779 (3)	171 (3)
O3—H3···O1^ii^	0.84 (3)	2.18 (3)	2.904 (3)	144 (4)

Symmetry codes: (i) *x*−1/2, −*y*+1/2, −*z*+2; (ii) *x*, *y*−1, *z*.
